# Dietary Iron Bioavailability: Agreement between Estimation Methods and Association with Serum Ferritin Concentrations in Women of Childbearing Age

**DOI:** 10.3390/nu10050650

**Published:** 2018-05-21

**Authors:** Eduardo De Carli, Gisele Cristina Dias, Juliana Massami Morimoto, Dirce Maria Lobo Marchioni, Célia Colli

**Affiliations:** 1 Department of Food and Experimental Nutrition, Faculty of Pharmaceutical Sciences, University of Sao Paulo, Sao Paulo 05508-000, Brazil; edecarli@usp.br (E.D.C.); gisele.dias@usp.br (G.C.D.); 2 Center for Biological and Health Sciences, Mackenzie Presbyterian University, Sao Paulo 01302-907, Brazil; juliana.morimoto@mackenzie.br; 3 Department of Nutrition, Faculty of Public Health, University of Sao Paulo, Sao Paulo 01246-904, Brazil; marchioni@usp.br

**Keywords:** iron status, algorithm, probabilistic approach

## Abstract

Predictive iron bioavailability (FeBio) methods aimed at evaluating the association between diet and body iron have been proposed, but few studies explored their validity and practical usefulness in epidemiological studies. In this cross-sectional study involving 127 women (18–42 years) with presumably steady-state body iron balance, correlations were checked among various FeBio estimates (probabilistic approach and meal-based and diet-based algorithms) and serum ferritin (SF) concentrations. Iron deficiency was defined as SF < 15 µg/L. Pearson correlation, Friedman test, and linear regression were employed. Iron intake and prevalence of iron deficiency were 10.9 mg/day and 12.6%. Algorithm estimates were strongly correlated (0.69≤ r ≥0.85; *p* < 0.001), although diet-based models (8.5–8.9%) diverged from meal-based models (11.6–12.8%; *p <* 0.001). Still, all algorithms underestimated the probabilistic approach (17.2%). No significant association was found between SF and FeBio from Monsen (1978), Reddy (2000), and Armah (2013) algorithms. Nevertheless, there was a 30–37% difference in SF concentrations between women stratified at extreme tertiles of FeBio from Hallberg and Hulthén (2000) and Collings’ (2013) models. The results demonstrate discordance of FeBio from probabilistic approach and algorithm methods while suggesting two models with best performances to rank individuals according to their bioavailable iron intakes.

## 1. Introduction

While iron deficiency persists as one of the most common nutritional disorders worldwide, which justifies the long-standing international efforts to increase iron intake of high-risk groups, a low dietary iron bioavailability (FeBio) is still presumed as the major cause of anemia (i.e., insufficient hemoglobin levels to meet body tissues demands for oxygenation) in both developed and developing countries [1]. Public health actions to address that issue include iron supplementation programs, food fortification policies, and nutritional counseling, especially targeted to children and women of childbearing age, notwithstanding the high daily iron requirements estimated for these two population groups [1,2]. 

However, even when an adequate amount of dietary/supplemental iron is provided, a high variability of iron absorption efficiency is expected among normal individuals [3,4]. It occurs mainly due to the inverse relationship between iron absorption and the size of body iron stores and because of the types of food, combinations of foods, and timings of food consumption, which jointly modulate the maximum extraction capacity of the mineral from the diet [3,5]. There are two forms of dietary iron: heme iron (exclusively found in animal tissues) and non-heme iron (widely distributed in foods, fortification, and/or supplementation compounds of elemental iron); heme iron is easily absorbed by humans [6]. Available non-heme iron, in turn, has a variable absorbability, even among individuals with no iron stores (1–45%), given the presence and the effect of its dietary absorption inhibitors (e.g., phytates, polyphenols, soy, and egg proteins) and enhancers (e.g., ascorbic acid, animal tissues, and alcohol) [3,5].

To comprehensively translate that complex relationship between iron intake and body iron status into practical tools to evaluate the adequacy of diets and public health interventions, a variety of iron absorption predictive models have been developed over the past 40 years [5,7,8,9,10,11,12,13,14]. These models included more than 8 different mathematical algorithms originally designed from iron labeled foods studies [3,5,7,8,13], in vitro dialysability assays [9], serum iron-curve assays [11], or meta-analysis of literature data [10,12]. Moreover, using data obtained from dietary surveys, at least four other algorithms were also proposed, as adaptations from existing models [15]. Despite the methodology particularities, a shared characteristic of all these equations is the summarization of mathematical adjustment terms for the effect of selected dietary elements on the iron availability from foods, meals, or complete diets [15,16,17]. However, only five out these models were derived from reliable isotopic or radioisotopic studies with biochemical characterization of meals/diets and included correction terms for the aforementioned effect of iron stores, besides specific dietary iron absorption enhancers and/or inhibitors [5,7,8,12,13]. Therefore, they allow the prediction of comparable FeBio estimates for individuals with varied dietary patterns and different assumed body iron status [5,7,8,12,13].

Monsen et al. (1978) were the first to propose an FeBio algorithm based on the intakes of heme iron and enhancers of non-heme iron absorption in meals [7]. Lately, other two meal-based algorithms were designed by Hallberg and Hulthén (2000) and Reddy et al. (2000), which also included adjustment terms for iron absorption inhibitors [5,8]. External studies that have subsequently attested the validity of these models are limited [15,16,17]. Two epidemiological trials assessing some of these methods noted inaccuracy and lack of agreement between them [18,19]. Moreover, there are criticisms regarding the feasibility of meal-based algorithms because they unsatisfactorily reflect the iron absorption adaptations occurring over time [17]. To overcome these limitations, Armah et al. (2013) and Colling et al. (2013) developed two new FeBio models based on complete diet data [12,13]. However, there is insufficient evidence to prove their accuracy and precision as well as their correlations with meal-based algorithms.

More recently, Dainty et al. (2014) described a probabilistic model to estimate the mean FeBio from populations with iron requirements and intake in a steady-state (for at least 1 year) using only individual measurements of the circulatory ferritin concentrations, a well-recognized body iron stores biomarker [20], and the usual total iron intake [14]. This approach generates a predictive FeBio value regardless of the errors associated with the quantification of heme iron and non-heme iron absorption inhibitors or enhancers [14,21]. However, since the steady-state body iron balance presumably takes long-term (as long as 12–36 months) to be established after great changes on bioavailable iron intake or requirement [22,23,24], the FeBio probabilistic approach is not applicable to children, pregnant, and lactating women [14]. The steady-state body iron balance may also be perturbed by frequent blood donations and immediate onset or interruption of menarche, menopause, hormonal contraceptive use, and iron supplements use [22,23,24].

In this study, having access to biochemical and dietary data of a selected group of healthy adult women at apparently steady-state body iron balance, we aim to prove the relative validity of currently published predictive FeBio mathematical models by assessing their correlation to each other and with serum ferritin (SF) concentrations. We hypothesized that the five tested different algorithms and the probabilistic approach models generate discordant FeBio estimates. However, we also expected that one or more of the algorithm estimates would be associated with women’s body iron stores, as measured by SF concentrations. Therefore, we assumed that, after adjustments for physiological losses, variations in steady-state body iron stores are potentially predicted by precise estimates of the usual bioavailable iron intake [25].

To allow comparability of the results, we adopted a definition of dietary FeBio consensually followed by scientific committees reporting reference values of dietary intakes, such as the United Nations Food and Agriculture Organization (FAO) and the Institute of Medicine (IOM) [26,27]. Hence, FeBio was defined as the percentage of the total ingested iron that is absorbed and utilized within the body by individuals with minimal iron stores (SF = 15 µg/L) [16,17].

## 2. Materials and Methods

### 2.1. Participants

This cross-sectional observational study was conducted on the main campus of the University of São Paulo (USP), São Paulo, Brazil, between May 2014 and June 2016. A sample size of 126 women was calculated prior to the start of the study, aiming a test power of 80% and a 2-tailed type I error rate of 5%, to detect significant differences of at least a half standard deviation on log-SF concentration values among women grouped by tertiles of bioavailable iron intake. Recruitment was conducted through posters, flyers, and electronic announcements. Volunteer students were screened using an eligibility questionnaire. If the initial criteria were met, these students were instructed to provide dietary intake information via a web-based system. Then, a blood collection, anthropometry measurements, and responding to general characterization questionnaires were scheduled. 

The exclusion criteria were as follows: (1) age < 18 or >45 years; (2) body mass index (BMI) < 17 or >30 kg/m^2^; (3) diagnosis of chronic disease, hemoglobinopathies, or hormonal dysfunction; (4) anemia (Hb < 12 g/dL), macrocytosis (VCM > 100 fl), microcytosis (VCM < 80 fl), or anisocytosis (RDW > 14.5%) in the absence of iron deficiency; (5) clinically important infection and/or inflammation (CRP >10 ng/dL); (6) alcohol abuse (>2 daily doses or >30 g/day); (7) smoking habit; (8) pregnancy or lactation in the last 2 years; (9) irregular menstrual cycle (interval < 21 or >35 days) in the last 2 years; (10) blood loss in the last 2 years due to accident, surgery, or blood donation; (11) diagnosis of intestinal parasites in the last 2 years; (12) intentional changes in eating habits in the last year; and (13) use of medication (except contraceptive) or vitamin-mineral supplement in the last year. Individuals with incomplete or invalid dietary data were also excluded from the study.

The study protocol was approved by the Research Ethics Committee of the Faculty of Pharmaceutical Sciences from the University of São Paulo (USP) (protocol number: 228.946/03-2013). All volunteers gave their written informed consent prior to participating in the study. Those with any hematological and/or biochemical abnormalities were referred for medical care.

### 2.2. Dietary Data Acquisition

Dietary intake was assessed via three web-based Food Records (FR) and an FFQ, using the online Nutriquanti system [28]. Briefly, the participants used an instruction manual attached to the electronic messages to access the system’s webpages and complete FRs on non-consecutive days, including one weekend day. Women were instructed to avoid changing their usual eating habits during the registration period. Moreover, they were encouraged to provide enough details about the types, portions, brands, and preparation modes of all food and beverages, based on a list with more than 3000 items. Meals were self-identified by participants.

Weights and volumes from household measures of all foods and beverages were standardized according to the national reference data [29,30]. Conversions of food into energy, iron, vitamin C, calcium, and alcohol intakes were mostly based on the national food chemical composition tables and [31,32], for few items, on an international database [33]. To add the food components required to estimate FeBio, the analytical data from our laboratory and other independent sources were used to ascribe heme iron [5,34,35,36], phytate [5,37], and polyphenols (tannic acid equivalents) to foods [5]. Moreover, based on the facts from food labels and standard recipe references [29,30], industrialized foods and composite dishes were broken down into ingredients to estimate the amounts of animal tissues, eggs, soy protein, and iron-fortified flours (corn and wheat). Unit conversions of phytate and raw animal tissue values to phytic acid (3.53 mg = 1 mg phytate phosphorus) and cooked tissue (1 g = 1.3 g raw tissue) were applied when necessary [5,7]. As proposed by Armah et al. (2013), tea and coffee equivalents (cups/day) were calculated by assuming that 1 cup of tea is equivalent to 2 cups (480 mL) of ice tea or 1.5 cup (360 mL) of coffee or herbal tea [13].

Invalid data in FRs were checked using the method proposed by McCrory et al. (2002) [38], assuming a cutoff limit of 2 standard deviations in the ratio between total energy intake (TEI) and total energy expenditure (TEE), as predicted by the equations from Vinken et al. (1999) [39]. Underreports were therefore considered as those whose mean TEI in FRs was lower than 50.8% of their predicted TEE. 

All participants with valid FRs were also requested to respond to an online version of a validated FFQ form for adults in São Paulo (SP) [40], which assessed the participants’ usual intake frequency of 84 foods, beverages, or food groups in the previous 12 months. 

### 2.3. Dietary Data Analysis

Using the online Multiple Source Method (MSM), daily means of all studied dietary variables were adjusted by the intraindividual data variability (deattenuation), to estimate the women’s usual intakes [41,42]. Data from the FFQ were used to define true non-consumers and usual consumer of animal tissues and alcoholic beverages, which served in MSM models as adjustment variable of daily heme iron and alcohol intake estimates, respectively. The same strategy was used to estimate the usual tea and coffee intake.

Before being employed in the analysis of correlation or stratification in quantiles, all dietary variables were adjusted by total energy intake values, using the residual method [43]. 

#### 2.3.1. Probabilistic Approach Estimate

Based on percentiles of iron physiological requirements described by the IOM for healthy adult women users and non-users of hormonal contraceptives [27], we constructed probability distributions of bioavailable dietary iron adequacy, varying in 5% increments, within a range of 0–100%, using the interpolation command of the online R program [44]. Then, these distributions were compared to 40 series of theoretical values of absorptions of the usual iron intake from each woman (range, 1–40%) to identify the probability that those different levels of bioavailable iron intake would meet the reference physiological iron requirements. Finally, the groups’ averages of iron inadequacy associated with each of the theoretical dietary iron absorption value was calculated. By approximation, the FeBio of each group was assumed to be the iron absorption value associated with a dietary inadequacy compatible with the prevalence of iron deficiency (SF < 15 μg/L) [45], given the observed usual total iron intake [14]. Finally, the weighted average of the two estimates separately made for user and non-user of hormonal contraceptives was calculated.

#### 2.3.2. Algorithm Estimates

Three meal-based algorithms [Monsen et al. (1978) [7], Hallberg and Hulthén (2000) [5], and Reddy et al. (2000) [8]] and two diet-based algorithms [Armah et al. (2013) [13], and Collings et al. (2013) [12]] were assessed. Selected characteristics of these five FeBio algorithms are summarized in Table 1. In all calculations, individual’s iron status was adjusted to 0 mg body store or a SF concentration equal to 15 μg/L (i.e., absence of iron stores), to estimate the maximum iron absorption from each individual diet and assess the sole influence of dietary factors [16]. 

Calculations were performed separately for heme and non-heme iron, but the sum of FeBio from the two fractions was used to characterize women’s diets (mg/day). While fixed values of heme FeBio were considered in Monsen et al. (1978) (35%), Reddy et al. (2000) (25%), Armah et al. (2013) (25%), and Collings et al. (2013) (25%) models, a specific equation was employed in Hallberg and Hulthén (2000) calculations. Non-heme iron was calculated as total iron discounted from heme iron. In all algorithms, 50% of the non-heme iron in mandatorily fortified wheat and corn flours from Brazil (4.2 mg of iron/100 g of flour) was not computed [46], since it is presumably not available for intestinal uptake. That assumption is based on the low relative bioavailability of elemental iron and insoluble iron salts [47], mostly employed by Brazilian mills for fortification purposes [46,48].

Previous studies have described some unrealistically non-heme FeBio estimates (>45%) when applying Hallberg and Hulthén (2000) and Reddy et al.’s (2000) algorithms to meals constituting high amounts of iron absorption enhancers [11,19,49]. Thus, when using these two models, meals with high vitamin C (>85 mg) and animal tissue (>175 g) contents had these values adjusted to 85 mg and 175 g (134.6 g of cooked tissues), respectively. In addition, non-heme FeBio estimates >45% were manually confined to this upper limit [49]. 

To calculate the dietary intake using Armah et al. (2013) and Collings et al.’s (2013) algorithms, complete diets were assumed as the daily food intake data from each of the women’s FRs. For the Collings et al. (2013) model, diets should be classified in three different types: “standard diet”, “with inhibitor”, and “with enhancer” [12]. Here, two classifications were considered: (1) women self-declared as vegetarians or meat restrictors were considered as having diets poor in iron absorption enhancers and/or rich in inhibitors (“with inhibitor”) and (2) omnivorous women was considered as having diets without modification (“standard diet”). 

Relative FeBio (%) were calculated as the ratio between deattenuated bioavailable iron and total iron intake values (mg/day).

### 2.4. Characterization Questionnaires and Anthropometry

Data regarding women’s sociodemographic, lifestyle, and menstrual flow information were collected using a self-administered questionnaire. Self-declared skin color/race was one of the five categories employed in the Brazilian census (“white”, “brown/mixed”, “black”, “yellow/Asian,” and “indigenous”). Socioeconomic level was inferred using the national criteria for scoring and stratification of social classes (A–E) [50]. Although vegetarianism or dietary meat restriction was self-declared, we checked the participants’ FRs and FFQ data for consistency. The short form of the International Physical Activity Questionnaire version 8 was applied to classify the physical activity levels (“sedentary”, “insufficiently active”, “active,” and “very active”) [51]. 

Based on the usual duration of periods as well as the types and amounts of commonly used tampons and/or pads, we calculated a validated score proposed by Heath et al. (1999) to stratify women according to tertiles of menstrual flow intensity. In addition, due to the great effect of hormonal contraceptives on menstrual iron losses (reduction of ~60%) and consequent long-term impact (up to 3 years) on the body iron balance [22,24,27], we carefully investigated the previous use of these medications or devices. Only women reporting no synthetic hormone use for at least 21 months in the last 2 years were considered contraceptive non-users. By contrast, women with a recent abandonment of these medicines were classified as hormonal contraceptive users if reporting a previous treatment of at least 18 months in the last 2 years.

Participants underwent anthropometric measurements three times in light clothing and with no shoes. Height was recorded to the nearest 0.1 cm using a portable stadiometer (Alturexata, Belo Horizonte, Brazil). Weights were recorded to the nearest 0.1 kg using an electronic digital platform scale (Kratos, Embu, Brazil). The mean values of height and weight were then used to calculate the BMI (BMI—kg/m^2^) and to diagnose undernutrition (<17 kg/m^2^), low or normal weights (17–24.9 kg/m^2^), overweight (24.9–29.9 kg/m^2^), and obesity (≥30 kg/m^2^) [52]. Moreover, waist circumference (WC) was measured to the nearest 0.1 cm at the midpoint between the lower margin of the last rib and the top of the iliac crest, using a retractable inelastic tape (AvaNutri, Três Rios, Brazil). High WC values served as an indicator of increased risk of cardiovascular and metabolic complications (>80 cm) [53].

### 2.5. Hematological and Biochemical Determinations

Women were requested to fast overnight for at least 8 h before a blood collection (08:00–11:00 a.m.). By venipuncture, blood samples were obtained in vacuum tubes containing EDTA (1.8 mg/µL), for hematological analysis, and in tubes with clot activator gel, for separation of serum by centrifugation at 4.000 RPM for 15 min. All blood analysis was performed by an accredited laboratory from the university hospital.

Complete blood count, including hemoglobin (Hb), hematimetric parameters, and reticulocyte count, was determined on the Sysmex XT-2000i equipment (GMI OpCo LLC., Minneapolis, MN, USA). Serum iron and total iron-binding capacity (CTLF) were determined using colorimetry and SF using direct chemiluminescence on the Advia Centaur XP equipment (Siemens AG., Munich, Germany). Transferrin saturation was calculated as the ratio of serum iron (μg/dL) to TIBC (μg/dL) multiplied by 100. High-sensitive C-reactive protein (CRP) and alfa1-acid glycoprotein (AGP) were determined using nephelometry on the BNII equipment (Siemens AG., Munich, Germany). The inter-assay coefficients of variability were 4.73%, 2.59%, and 5.81% for serum iron, CTLF, and SF determinations, respectively.

It has been long recognized that inflammation can increase SF, therefore affecting the assessment of iron status [45,54]. Hence, before any analysis, we corrected the SF concentration values by a possible subclinical infection and/or inflammation, as proposed by Thurnham and McCabe (2012) [54]. CRP > 5 mg/L and AGP > 100 mg/dL indicated the acute phases of incubation (for increased CRP only), early convalescence (for increased AGP and CRP), or late convalescence (for increased AGP only), for which the specific correction factors of 0.77, 0.53, or 0.75 on SF concentration values were applied, respectively [54]. 

Iron deficiency was diagnosed as SF < 15 μg/L [45], and iron-deficient erythropoiesis as transferrin saturation < 16% [2,27]. Iron overload were defined as SF > 150 μg/L and transferrin saturation > 60% [2,45]. Anemia was diagnosed when Hb < 12 g/dL [1,2].

### 2.6. Statistical Analysis

Analysis was performed using IBM SPSS (version 20.0, IBM SPSS Inc., Chicago, IL, USA) and GraphPad Prism (version 5.0, GraphPad Software Inc., San Diego, CA, USA) software. A significance level adopted in all statistical tests was 5%.

The normal distribution of continuous variables was evaluated using Kolmogorov-Smirnov test. When necessary, natural logarithm transformation was applied on variable values to approximate normal distributions. Data are presented as means and standard deviations or geometric means and 95% confidence intervals. 

Due to the heteroscedasticity between values distributions, repeated measurements from algorithms (mg/day and %) were compared using the non-parametric Friedman test, followed by Dunn’s post hoc test. The linear association between continuous variables was assessed using Pearson’s correlation and simple linear regression analysis. To compare the independent groups, chi-square test, likelihood ratio test, or Fisher’s tests were used to analyze categorical variables, and Student’s t-test or analysis of variance (ANOVA) followed by polynomial contrast for linear trends or Tukey’s post hoc test for continuous variables.

Multiple linear regression was used to test the associations between SF and tertiles or combined extreme quartiles of the dietary variables. The covariates included in the multiple models were age (years), BMI (kg/m^2^), hormonal contraceptive use (yes vs. no), menstrual flow score (tertiles), self-declared skin color/race (white vs. black or brown/mixed; white vs. yellow), and physical activity levels (active vs. insufficiently active; active vs. very active). In sensitive analysis, an additional adjustment for self-declared vegetarianism or dietary meat restriction (yes vs. no) was also tested. In all models, possible interactions between dietary variables and hormonal contraceptive use were evaluated. Residual analysis and collinearity diagnosis were performed to assess the validity of the final regression models.

## 3. Results

### 3.1. Participants’ Characteristics

**Figure 1:**
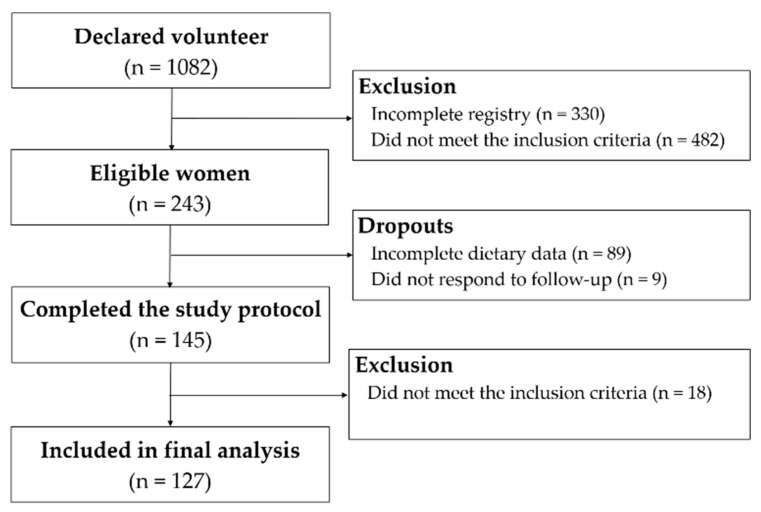
Study flow diagram.

The study flow diagram is shown in Figure 1. One hundred and twenty-seven students were included in the final analysis. Mean age was 27 years, and mean body mass index was 22 kg/m^2^. Participants who were considered obese were not recruited; however, 26% of women had high WC and 11% was overweight. According to the national criteria, >80% of the students belonged to upper-middle or upper socioeconomic classes (A–B). About three-quarters of the sample were self-declared as white (74.8%) and two-thirds were classified as physically active or very active (61.4%) (Table 2). Non-omnivores included 10 lacto-ovo-vegetarians (7.9%), 6 pesco-vegetarians (4.7%), and 2 vegans (1.6%). 

None of women reported the use of intrauterine devices. In contrast, 60% of women reported the use of hormonal contraceptives, which was more frequent among women <30 years old (*p <* 0.05) and those belonging to the upper-middle socioeconomic class (*p <* 0.05). These women showed significantly lower levels of menstrual flow intensity score (*p <* 0.01) and, curiously, a higher prevalence of subclinical inflammation (CRP > 5 mg/L) (Table 2). 

Mean total iron intake was estimated to be 10.9 mg/day, which is approximately 6% of heme iron and 29% of iron from fortified corn and wheat flours (Table 2). Lunch and dinner showed comparable total daily iron amounts (3.71 mg/day and 3.12 mg/day), although differed in almost 50% on their contributions to total daily heme iron intake (62.8% and 33.0%). In turn, breakfast and snacks provided the highest daily amounts of calcium (32.6% and 25.3%) and polyphenols (32.9% and 47.9%) (Table S1).

Geometric mean of SF concentrations was 36.6 μg/L. While no iron overload case was found, iron deficiency was diagnosed in 12.6% of women, one-quarter of whom were also anemic. Even though hormonal contraceptive users had a slightly higher mean total iron intake than non-users (10.3 mg/day vs. 11.2 mg/day, *p <* 0.05), iron deficiency was three times more prevalent among the latter (6.7% vs. 21.2%, *p <* 0.05), in accordance with their evident differences in menstrual iron losses (Table 2).

### 3.2. Dietary Iron Bioavailability Estimates

**Figure 2:**
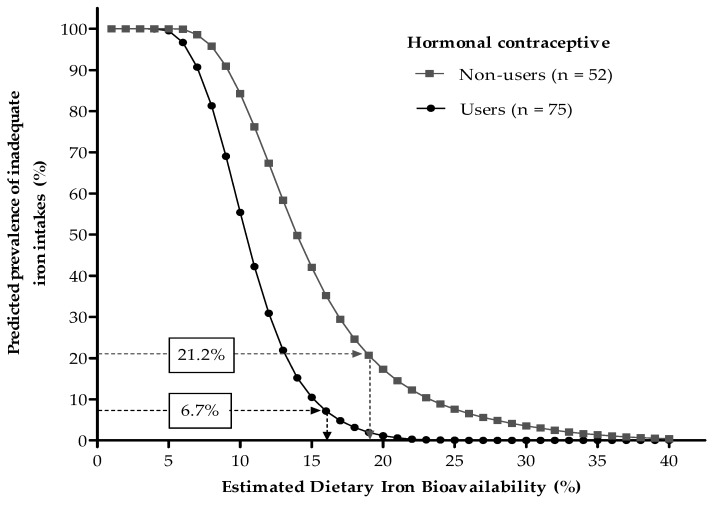
Predicted prevalence of inadequate iron intakes at different dietary iron bioavailabilities, according to the probabilistic approach proposed by Dainty et al. [14]. Values in boxes are prevalence of iron deficiency (serum ferritin < 15 µg/L). Mean dietary iron bioavailabilities of 16% and 19% were estimated for hormonal contraceptive users and non-users, respectively.

As illustrated in Figure 2, based on physiologic iron requirement distributions, usual total iron intakes, and prevalence of iron deficiency among hormonal contraceptive users and non-users, their expected dietary FeBio values were of 16% and 19%, respectively (Figure 2). A weighted average of these two estimates, assumed as a mean for the overall sample, was calculated as 17.2%. No substantial changes in this value were observed after excluding vegetarians or meat restrictors from the analysis.

Table 3 shows the estimates from the five tested algorithms. Although 1.7% and 4.1% of meals analyzed using Hallberg and Hulthén (2000) and Reddy et al.’s (2000) models required manual confinement of unrealistic high non-heme iron absorption values (>45%), their dietary FeBio estimates (12.02% and 12.80%, respectively) did not differ from those of Monsen et al.’s (1978) model (11.57%), which was also based on meals. By contrast, significantly lower bioavailability values were obtained using the diet-based algorithms of Armah et al. (2013) and Collings et al. (2013) (8.91% and 8.51%, respectively). 

Upon searching for dietary variables related to each of the tested algorithm estimates, we found all of them were strong negatively associated with heme iron and animal tissue intakes. Only estimates from Hallberg and Hulthén (2000) and Reddy et al.’s (2000) algorithms were unrelated to non-heme iron, while were negatively correlated with dietary phytate intakes. A negative association was also found between calcium and the former algorithm estimates. In turn, only Reddy et al. (2000) and Collings et al.’s (2013) models provided estimates that independently varied from dietary vitamin C intakes (Table S2).

### 3.3. Correlation between Dietary Iron Bioavailability Estimates 

Notably, all FeBio algorithms underestimated 25%–50% of the probabilistic model; all algorithm estimates, whether based on meals or complete diets, were strongly correlated. However, Collings et al.’s (2013) model showed a weaker association with meal-based measurements when compared to Armah et al.’s (2013) model. The highest correlations were found between Hallberg and Hulthén (2000) and Reddy et al. (2000) models (r = 0.851, *p <* 0.001) and between Monsen et al. (1978) and Armah et al.’s (2013) models (r = 0.851, *p <* 0.001). No slope coefficients from regression equations between algorithm estimates was close to 1 (0.8 ≥ β ≥ 1.2), suggesting a lack of identity between them (Table 4).

### 3.4. Association between Dietary Iron Bioavailability Estimates and Serum Ferritin Concentrations

**Figure 3:**
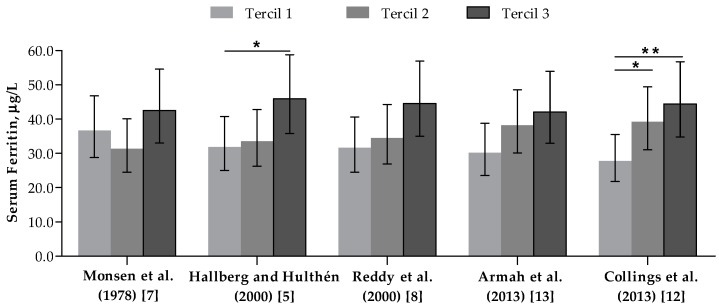
Serum ferritin (µg/L) of women classified according to tertiles of bioavailable iron intake (mg/day). *n* = 127. Bars and whiskers indicate geometric mean and 95% confidence intervals, respectively. * *p <* 0.05, ** *p <* 0.01—in comparison to the first tertile, according to multiple linear regression adjusted for age, body mass index, skin color/race, physical activity level, hormonal contraceptives use and menstrual flow intensity level.

SF concentrations according to FeBio estimates are presented in Figure 3. No significant association was found between the iron biomarker and FeBio measured with Monsen et al. (1978), Reddy et al. (2000), or Armah et al. (2013) algorithms. In contrast, significant differences of 30.5% and 37.4% were estimated in multiple-adjusted geometric means of SF between women stratified by extremes tertiles of FeBio using Hallberg and Hulthén (2000) (*p <* 0.05) and Collings et al.’s (2013) algorithm (*p <* 0.01), respectively. Moreover, differences in SF concentration values of approximately 30% was found among women classified based on the median values of these two algorithm measurements (*p <* 0.05 and *p <* 0.01, respectively) (Figure S1). 

To assess a possible mediation effect of the non-omnivorous dietary pattern on the associations between algorithm measurements and SF concentrations values, an additional adjustment for vegetarians or meat restrictors in regression models was tested. In this sense, the association between SF and tertiles of FeBio measured using Collings et al.’s (2013) model, but not with Hallberg and Hulthén (2000) model, remained statistically significant (*p* = 0.021 and *p* = 0.103, respectively). Interestingly, only Hallberg and Hulthén’s (2000) algorithm estimates were also associated with transferrin saturation (Table S3).

Besides FeBio measures, quantiles of total iron, non-heme iron, and heme iron intakes were also evaluated in relation to SF, but no significant association was found. Moreover, neither of the tested regression models showed an interactive effect between dietary variables and hormonal contraceptive use on the biomarker concentrations.

## 4. Discussion

Using the dietary and biochemical data from a selected group of healthy women of childbearing age and steady-state body iron balance, we demonstrated that FeBio values from a probabilistic approach contradict those from predictive algorithms, especially those complete diet-based models. Still, when searching for estimates that could predict SF concentrations, we found one meal-based (Hallberg and Hulthén (2000) [5]) and one diet-based (Collings et al. (2013) [12]) methods that can better rank individuals according to their bioavailable iron intakes. From our knowledge, this is the first study to compare the different FeBio models and evaluate their practical usefulness using a set of data typically available in cross-sectional epidemiologic surveys.

Aimed at studying heathy women with reliable dietary and biochemical data, extensive selection criteria were used in screening the volunteers, which may have biased the inclusion of a relatively iron-sufficient population (87.4%). This scenario may have increased the degree of uncertainty on the FeBio estimate from the probabilistic model [14], as evidenced by a 3% difference on FeBio estimates for hormonal contraceptive users and non-users. These two groups differed in approximately 1 mg/day on the usual total iron intakes and in almost 10 times on the prevalence of subclinical inflammation, which possibly affected the dietary and biochemical inadequacy estimates. However, it is unlikely that this small iron intake difference has substantially impacted our results, and we corrected the SF to remove the effect of inflammation on this iron biomarker [54]. Additionally, our overall mean FeBio estimated with this model (17.2%) was very close to that assumed for diverse Western diets (18%) [27], providing an initial evidence of good performance to this method. Further studies with populations at higher risk of iron deficiency might result in more accurate FeBio calculations using the probabilistic approach. 

To assess the relative validity of different FeBio methods, we then compared the estimates from the five algorithms to each other and with the probabilistic approach, assuming the latter model is the least affected by measurement errors associated with collection and analysis of dietary data [14]. Based on our results, all algorithms underestimated the probabilistic approach; however, the highest discordance from all other tested models were observed for the diet-based algorithms of Collings et al. (2013) and Armah et al. (2013) [12,13]. It surprisingly occurred even though these two models were published as presumably more accurate alternatives to the traditional meal-based algorithms [12,13]. These findings indicate a possible exaggerated effect attributed to SF concentrations or to dietary elements on the non-heme FeBio using these two aforementioned methods. 

Our results largely differed from those described by Armah et al. (2015) and Perignon et al. (2018) who, using the algorithm proposed by the former researchers [13], estimated higher FeBio means in the diet of North Americans (15%) and French (13%), respectively [55,56]. Possibly, our assumption of a large unavailability for uptake to the non-heme iron from Brazilian-fortified flours (50%) and dietary dissimilarities of our studied women have contributed to these discordances [46,47]. Nevertheless, the precision and accuracy of all models tested in our study have not yet been extensively explored by external studies. Hence, our results suggest that FeBio values estimated with algorithms must be carefully employed when used as a basis for readjusting dietary references values of iron intake or when applied in public health policies aimed at treating anemia.

In fact, two previous studies that tested the accuracy of FeBio algorithms described similar results [18,19]. In a cohort of religious women from the Philippines, Beard et al. (2007) reported that the algorithms of Monsen et al. (1978), Hallberg and Hulthén (2000), and Reddy et al. (2000) underestimated 57–66% of the FeBio values objectively predicted from the women’s change in body iron stores following the introduction of a high iron rice variety in their diets [19]. In a second study, Zimmermann et al. (2005), also based on an indirect calculation of FeBio from changes in the body iron status of Moroccan children, found a 50% underestimation for measurements obtained with the Reddy et al. (2000) algorithm [18].

Based on our findings, Hallberg and Hulthén (2000) and Collings et al.’s (2013) models provided FeBio estimates that independently predicted the SF variability among the studied women [5,12]. Moreover, those estimates obtained using Collings et al.’s (2013) algorithm—the simplest of all the tested models—surprisingly had a relatively stronger association with this biomarker. Although non-identical estimates were provided, these two models were strongly correlated, despite their deep methodologic particularities. The Hallberg and Hulthén (2000) algorithm—the most refined and complex of all published FeBio models—was elaborated from a set of radioisotope assays testing iron absorption from chemical characterized meals. It exhibits enhancing and inhibitory effects on the dietary elements by means of eight equations applicable to the non-heme iron and one to the heme iron intakes. In addition, it includes correction terms for the efficiency of both heme and non-heme iron absorptions by the SF concentrations, using exponential equations [5]. The Collings et al.’s (2013) model, in turn, was generated from a meta-analysis of radioisotope or stable isotope assays testing complete diets (food intake periods ≥ 1 day). It also employs exponential equation for correction of non-heme iron absorption by SF concentrations but lack an adjustment term to heme iron absorption. Exceptionally, this model does not require any quantitative dietary data for its calculations, except the usual non-heme iron intake. Its equation is summarized in three constant bioavailability values applied in function of the diet under analysis, arbitrarily classified according to its overall content of inhibitors or enhancers of iron absorption [12].

Hence, we found significant associations between dietary enhancers and inhibitors of iron absorption with FeBio estimates from Hallberg and Hulthén’s (2000) model, but not from Collings et al.’s (2013) model. Thus, important dietary variations possibly captured by Collings et al.’s (2013) algorithm, but not by Hallberg and Hulthén (2000) algorithm, are not likely the cause of the relatively stronger association between SF and estimates from the first model. Nevertheless, it is reasonable to assume that these two algorithms were the most efficient of the tested models due their relatively more appropriate adjustments terms ascribed to dietary factors on the non-heme iron absorption. In this sense, we found that the Hallberg and Hulthén’s (2000) algorithm better discriminated FeBio differences between omnivorous and vegetarian diets and was the unique model significantly associated with women’s transferrin saturation values, besides the SF. Taken together, our findings confirm Hunt’s (2010) observations that, in an attempt to validate different meal-based FeBio algorithms testing omnivorous and vegetarian diets, showed best results for the Hallberg and Hulthén’s (2000) model [17].

The limitation of our study is the small sample size with possible low representativeness of the general population. Moreover, we collected dietary data using FRs, which are subjected to reactivity, leading to changes in food intake or food reporting [57]. In this sense, however, our dietary estimates indicated a mean total iron intake (10.9 mg/day) very close to that described for Brazilian adult women in a national dietary survey (10.1 mg/day) [58]. Similarities were found in the contribution of flour fortification (~30%) to the total iron intake [59], and in the mean values of total animal tissue intake (130.7 g/day of cooked tissues) [60]. The estimated phytate contents in the evaluated meals were also very similar to those found in directly analyzed samples designed to reflect the Brazilian pattern [61], despite the use of international food phytate databases [5,37]. Nevertheless, our findings must be confirmed, especially in larger population-based studies, by using not reactive diet assessment methods (e.g., 24-h food recall) and by employing data from chemical analysis of national foods.

In our experimental conditions, neither total iron, non-heme iron, nor heme intakes were strongly correlated with SF as were FeBio estimates. These observations reinforce the importance of considering the effect of dietary modifiers of iron absorption for a better interpretation of the role of diet on body iron status and its related health or disease outcomes. Taken together, our findings signal the need for validation of currently available FeBio models for use in epidemiological dietary surveys. Based on the data presented herein, the original hypothesis that predictive mathematical algorithms and the probabilistic approach models generate discordant FeBio estimates may be accepted. Also, we highlight that, among women of childbearing age and varied dietary patterns, Hallberg and Hulthén (2000) and Collings’ (2013) algorithms showed the best results at predicting body iron statuses, as measured by SF concentrations. 

## Figures and Tables

**Table 1:** Summary characteristics of the five tested iron bioavailability algorithms.

Algorithm Model	Calculus Basis	Dietary Factors Adjusting Non-Heme Iron Absorption	Dietary Factors Adjusting Heme Iron Absorption	Adjustment Made for Individual’s Body Iron Status
Monsen et al. (1978) [7]	Meals	Cooked animal tissues Vitamin C	None	Heme and nonheme iron absorption are corrected for the body iron stores of 0, 250, 500, and 1000 mg, using specific correction factors.
Hallberg and Hulthén (2000) [5]	Meals	Raw animal tissues Vitamin C Alcohol Phytate phosphorous Calcium Polyphenols Soy Protein Eggs	Calcium	Heme and nonheme iron absorptions are corrected according to serum ferritin concentration, using two independent equations.
Reddy et al. (2000) [8]	Meals	Cooked animal tissues Vitamin C Phytic acid	None	Nonheme iron absorption is corrected according to serum ferritin concentration, using an equation proposed by Cook [3]. Lacks a correction term for heme iron absorption. A fixed factor of 25% of heme iron absorption was assumed, as proposed by the Institute of Medicine [27].
Armah et al. (2013) [13]	Complete diets	Cooked animal tissues Vitamin C Phytic acid Calcium Tea and coffee equivalents Non-heme iron	None	Nonheme iron absorption is corrected according to serum ferritin concentration, using an equation incorporate in the same model of adjustments for dietary factors. Lacks a correction term for heme iron absorption. A fixed factor of 25% of heme iron absorption was assumed, as proposed by the Institute of Medicine [27].
Collings et al. (2013) [12]	Complete diets	Diets are categorized into 3 types: - Self-selected (standard diet) - With inhibitors (high calcium, low vitamin C, no meat) - With enhancers (low calcium, high vitamin C, high meat)	None	Nonheme iron absorption is corrected according to serum ferritin concentration, using an equation incorporate in the same model of adjustments for dietary factors. Lacks a correction term for heme iron absorption. A fixed factor of 25% of heme iron absorption was assumed, as proposed by the Institute of Medicine [27].

**Table 2:** General characterization of women at presumably steady-state body iron balance.

Selected Characteristics	Overall Sample	Hormonal Contraceptives	
		Non-users	Users
	(*n* = 127)	(*n* = 52)	(*n* = 75)
**Sociodemographic and lifestyle characterization**			
Age (years)	27.4 (5.1) [18.0–42.0]	27.8 (6.0)	27.2 (4.3)
>30 year, n (%)	34 (26.8)	19 (36.5)	15 (20.0) ^#^
Body Mass Index (kg/m^2^)	22.1 (2.4) [17.0–29.1]	21.8 (2.4)	22.2 (2.4)
>25 Kg/m^2^, n (%)	14 (11.0)	5 (9.6)	9 (12.0)
Waist circumference (cm)	75.4 (7.1) [60.5–99.3]	75.9 (7.1)	75.5 (7.1)
>80 cm, n (%)	33 (26.0)	15 (28.8)	18 (24.0)
Self-declared skin color/race, n (%)			
White	95 (74.8)	36 (69.2)	59 (78.7)
Black or brown/mixed	24 (18.9)	11 (21.2)	13 (17.3)
Yellow/Asian	8 (6.3)	5 (9.6)	3 (4.0)
Socioeconomic level, n (%) *			
Class A	15 (12.0)	6 (11.5)	9 (12.3)
Class B	86 (68.8)	30 (57.7)	56 (76.7) ^#^
Classes C or D	24 (19.2)	16 (30.8)	8 (11.0) ^#^
Self-declared diet, n (%)			
Omnivorous	109 (85.8)	43 (82.7)	66 (88.0)
Vegetarians or meat restrictors	18 (14.2)	9 (17.3)	9 (12.0)
Physical Activity Level, n (%)			
Active	62 (48.8)	22 (42.3)	40 (53.3)
Very active	16 (12.6)	7 (13.5)	9 (12.0)
Insufficiently active	49 (38.6)	23 (44.2)	26 (34.7)
**Menstrual flow characterization**			
Periods duration, n (%)			
2–3 days	20 (15.7)	6 (11.5)	14 (18.7)
4–5 days	89 (70.1)	37 (71.2)	52 (69.3)
6–7 days	18 (14.2)	9 (17.3)	9 (12.0)
Menstrual flow intensity score, n (%)			
Tertile 1 (≤13 units)	44 (34.6)	14 (26.9)	30 (40.0) ^##^
Tertile 2 (14–21 units)	39 (30.7)	12 (23.1)	27 (36.0) ^##^
Tertile 3 (≥22 units)	44 (34.6)	26 (50.0)	18 (24.0) ^##^
**Hematological and biochemical characterization**			
Hemoglobin (g/dL)	13.4 (0.8) [11.0–15.4]	13.4 (0.9)	13.4 (0.8)
<12 g/dL, n (%)	4 (3.1)	3 (5.8)	1 (1.3)
Transferrin saturation (%)	29.9 (11.4) [6.4–59.7]	30.1 (12.8)	29.8 (10.3)
<16%, n (%)	14 (11.0)	7 (13.5)	7 (9.3)
Ferritin (µg/L) **	36.6 (31.4; 42.6) [4.0–198.0]	31.0 (23.6; 40.7)	41.0 (34.3; 49.0)
<15 µg/L, n (%)	16 (12.6)	11 (21.2)	5 (6.7) ^#^
Alfa1-Acid Glycoprotein (mg/dL)	61.4 (18.9) [26.0–133.0]	65.6 (16.4)	71.9 (20.8)
≥100 mg/dL, n (%)	4 (3.1)	2 (3.8)	2 (2.7)
High-sensitive C-Reactive Protein (mg/L)	1.1 (0.9; 1.3) [0.2–9.8]	0.5 (0.4; 0.7)	1.7 (1.4; 2.3) ^##^
≥5 mg/L, n (%)	15 (11.8)	1 (1.9)	14 (18.7) ^##^
**Dietetic characterization**			
Total energy (kcal/day)	2032.5 (436.5) [958.0–3531.9]	1984.6 (465.5)	2065.7 (415.1)
Total iron (mg/day)	10.9 (2.2) [6.1–19.5]	10.3 (2.2)	11.2 (2.2) ^#^
Non-heme iron (mg/day)	10.2 (2.2) [5.8–18.1]	9.6 (2.1)	10.5 (2.2) ^#^
Fortification iron (mg/day)	3.2 (1.2) [0.4–6.3]	2.9 (1.3)	3.4 (1.1) ^#^
Heme iron (mg/day)	0.7 (0.4) [0.0–2.1]	0.7 (0.5)	0.7 (0.4)
Animal tissues (g/day) ^1^	169.9 (76.8) [0.0–325.2]	172.1 (79.6)	168.4 (75.2)
Vitamin C (mg/day)	129.0 (81.9) [20.5–434.1]	121.1 (65.8)	134.5 (91.5)
Calcium (mg/day)	845.1 (285.8) [210.3–1872.1]	844.6 (2851)	845.5 (288.2)
Phytate (mg/day) ^2^	260.2 (84.1) [82.0–563.5]	246.7 (77.7)	269.5 (87.5)
Polyphenols (mg/day) ^3^	223.3 (82.7) [56.4–563.0]	234.1 (94.1)	215.8 (73.4)
Tea and coffee equivalents (cups/day) ^4^	0.3 (0.3; 0.4) [0.0–2.0]	0.4 (0.3; 0.5)	0.3 (0.2; 0.4)
Alcohol (g/day)	1.1 (1.0; 1.3) [0.0–11.3]	1.1 (0.9; 1.4)	1.1 (1.0; 1.3)

Values are mean (SD), geometric mean (95% CI) or absolute count (percentage). Ranges for continuous variables are listed in brackets. * Data missing for two participants. ** Inflammation-corrected SF values were transformed into natural logarithm before analysis. ^1^ Raw tissue. ^2^ Phytate phosphorous. ^3^ Tannic acid equivalents. ^4^ As proposed by Armah et al. (2013) [13]. ^#. ##^ Significant difference between hormonal contraceptive users and non-users according to *t*-Student’s test (continuous variables) or Chi-Squared, *Likelihood Ratio* or *Fisher* tests (categorical variables). ^#^ *p <* 0.05; ^##^ *p <* 0.01.

**Table 3:** Dietary iron bioavailability (FeBio) estimates according to meal-based and diet-based algorithms.

Algorithm Models	Absolute FeBio (mg/day)	Relative FeBio (%)
Monsen et al. (1978) [7]	1.26 (0.36) [0.45–2.44] ^a^	11.57 (2.21) [6.54–17.95] ^a^
Hallberg and Hulthén (2000) [5]	1.30 (0.42) [0.44–2.88] ^a^	12.02 (3.34) [4.10–24.90] ^a^
Reddy et al. (2000) [8]	1.38 (0.48) [0.40–3.41] ^a^	12.80 (3.87) [3.61–25.07] ^a^
Armah et al. (2013) [13]	0.96 (0.21) [0.49–1.67] ^b^	8.91 (1.36) [5.62–13.33] ^b^
Collings et al. (2013) [12]	0.92 (0.23) [0.49–1.84] ^b^	8.51 (1.04) [4.61–11.44] ^b^

*n* = 127. Values are mean (SD). Ranges are listed in brackets. Friedman’s test indicates significant differences between estimates of both absolute and relative iron bioavailability (*p <* 0.001). ^a, b^ Different subscribed letters in a same column indicate statistical difference between algorithm models, according to Dunn’s *post hoc* test.

**Table 4:** Correlation coefficients and simple linear regression equations of the bivariate relationship between iron bioavailability estimates (mg/day).

Algorithm Models	*Monsen et al. (1978)* [7]	*Hallberg and Hulthén (2000)* [5]	*Reddy et al. (2000)* [8]	*Armah et al. (2013)* [13]
Hallberg and Hulthén (2000) [5]	r = 0.823 y = 1.5x − 0.8			
Reddy et al. (2000) [8]	r = 0.718 y = 1.7x − 0.9	r = 0.851 y = 1.2x + 0.0		
Armah et al. (2013) [13]	r = 0.850 y = 0.6x + 0.1	r = 0.836 y = 0.4x + 0.4	r = 0.749 y = 0.3x + 0.4	
Collings et al. (2013) [12]	r = 0.793 y = 0.5x + 0.1	r = 0.764 y = 0.3x + 0.4	r = 0.694 y = 0.3x + 0.4	r = 0.809 y = 0.8x + 0.1

*n* = 127. Pearson’s correlation test was used. All models had *p <* 0.001.

## Supplementary Materials

The following are available online at http://www.mdpi.com/2072-6643/10/5/650/s1, Table S1: Selected dietary characteristics according to meals in the diet of women at presumably steady-state body iron balance; Table S2: Selected dietary characteristics according to quantiles of bioavailable iron intake (mg/day) of women at presumably steady-state body iron balance; Table S3: Selected hematological and biochemical characteristics according to quantiles of bioavailable iron intake (mg/day) of women at presumably steady-state body iron balance. Figure S1. Serum ferritin (µg/L) of women stratified at median of bioavailable iron intake (mg/day).

## References

[1] World Health Organization (2017). Nutritional Anaemias: Tools for Effective Prevention and Control.

[2] World Health Organization (2001). Iron Deficiency Anaemia: Assessment, Prevention and Control. A Guide for Programme Managers.

[3] Cook J.D., Dassenko S.A., Lynch S.R. (1991). Assessment of the role of nonheme-iron availability in iron balance. Am. J. Clin. Nutr..

[4] Hoppe M., Hulthén L., Hallberg L. (2008). The importance of bioavailability of dietary iron in relation to the expected effect from iron fortification. Eur. J. Clin. Nutr..

[5] Hallberg L., Hulthén L. (2000). Prediction of dietary iron absorption: An algorithm for calculating absorption and bioavailability of dietary iron. Am. J. Clin. Nutr..

[6] Hallberg L., Hulthén L., Gramatkovski E. (1997). Iron absorption from the whole diet in men: How effective is the regulation of iron absorption?. Am. J. Clin. Nutr..

[7] Monsen E.R., Hallberg L., Layrisse M., Hegsted D.M., Cook J.D., Mertz W., Finch C.C. (1978). Estimation of available dietary iron. Am. J. Clin. Nutr..

[8] Reddy M.B., Hurrell R.F., Cook J.D. (2000). Estimation of nonheme-iron bioavailability from meal composition. Am. J. Clin. Nutr..

[9] Chiplonkar S.A., Agte V.V. (2006). Statistical model for predicting non-heme iron bioavailability from vegetarian meals. Int. J. Food Sci. Nutr..

[10] Conway R.E., Powell J.J., Geissler C.A. (2007). A food-group based algorithm to predict non-heme iron absorption. Int. J. Food Sci. Nutr..

[11] Rickard A.P., Chatfield M.D., Conway R.E., Stephen A.M., Powell J.J. (2009). An algorithm to assess intestinal iron availability for use in dietary surveys. Br. J. Nutr..

[12] Collings R., Harvey L.J., Hooper L., Hurst R., Brown T.J., Ansett J., King M., Fairweather-Tait S.J. (2013). The absorption of iron from whole diets: A systematic review. Am. J. Clin. Nutr..

[13] Armah S.M., Carriquiry A., Sullivan D., Cook J.D., Reddy M.B. (2013). A Complete Diet-Based Algorithm for Predicting Nonheme Iron Absorption in Adults. J. Nutr..

[14] Dainty J.R., Berry R., Lynch S.R., Harvey L.J., Fairweather-Tait S.J. (2014). Estimation of Dietary Iron Bioavailability from Food Iron Intake and Iron Status. PLoS ONE.

[15] Reddy M.B. (2005). Algorithms to assess non-Heme Iron Bioavailability. Int. J. Vitam. Nutr. Res..

[16] Lynch S. (2005). The Precision of in vitro Methods and Algorithms for Predicting the Bioavailability of Dietary Iron. Int. J. Vitam. Nutr. Res..

[17] Hunt J.R. (2010). Algorithms for Iron and Zinc Bioavailability: Are they Accurate?. Int. J. Vitam. Nutr. Res..

[18] Zimmermann M.B., Chaouki N., Hurrell R.F. (2005). Iron deficiency due to consumption of a habitual diet low in bioavailable iron: A longitudinal cohort study in Moroccan children. Am. J. Clin. Nutr..

[19] Beard J.L., Murray-Kolb L.E., Haas J.D., Lawrence F. (2007). Iron Absorption Prediction Equations Lack Agreement and Underestimate Iron Absorption. J. Nutr..

[20] Cook J.D., Lipschitz D.A., Miles L.E.M., Finch C.A. (1974). Serum ferritin as a measure of iron stores in normal subjects. Am. J. Clin. Nutr..

[21] Fairweather-Tait S.J., Jennings A., Harvey L.J., Berry R., Walton J., Dainty J.R. (2017). Modeling tool for calculating dietary iron bioavailability in iron-sufficient adults. Am. J. Clin. Nutr..

[22] Hultén L., Gramatkovski E., Gleerup A., Hallberg L. (1995). Iron absorption from the whole diet. Relation to meal composition, iron requirements and iron stores. Eur. J. Clin. Nutr..

[23] Hallberg L., Hulthén L., Garby L. (1998). Iron stores in man in relation to diet and iron requirements. Eur. J. Clin. Nutr..

[24] Hallberg H., Hulthén L., Garby L. (2000). Iron stores and haemoglobin iron deficits in menstruating women. Calculations based on variations in iron requirements and bioavailability of dietary iron. Eur. J. Clin. Nutr..

[25] Hallberg L. (2000). New tools in studies on iron nutrition. Principles, applications and consequences. Scand. J. Food Nutr..

[26] Food and Agriculture Organization of the United Nations (2001). Human Vitamin and Mineral Requirements.

[27] Institute of Medicine (US) Panel on Micronutrients (2001). Dietary Reference Intakes for Vitamin A, Vitamin K, Arsenic, Boron, Chromium, Copper, Iodine, Iron, Manganese, Molybdenum, Nickel, Silicon, Vanadium, and Zinc.

[28] Galante A. (2007). Desenvolvimento e validação de um método computadorizado para avaliação do consumo alimentar, preenchido por indivíduos adultos utilizando a Web. Ph.D. Thesis.

[29] Fisberg R.M., Villar B.S. (2002). Manual de Receitas e Medidas Caseiras para Cálculo de Inquéritos Alimentares.

[30] Pinheiro A.B.V. (2008). Tabela para Avaliação de Consumo Alimentar em Medidas Caseiras, 5th ed.

[31] NEPA—UNICAMP (2011). Tabela Brasileira de Composição de Alimentos.

[32] Universidade de São Paulo. Faculdade de Ciências Farmacêuticas, Departamento de Alimentos e Nutrição Experimental/BRASILFOODS (1998). Tabela Brasileira de Composição de Alimentos-USP.

[33] US Department of Agriculture, Agricultural Research Service, Nutrient Data Laboratory (2015). USDA National Nutrient Database for Standard Reference.

[34] Kongkachuichai R., Napatthalung P., Charoensiri R. (2002). Heme and Nonheme Iron Content of Animal Products Commonly Consumed in Thailand. J. Food Compost. Anal..

[35] Schönfeldt H.C., Hall N.G.H. (2011). Determining iron bio-availability with a constant heme iron value. J. Food Compost. Anal..

[36] Mistura L.P.F. (2006). Cinética de ruptura do ferro heme em carne bovina (coxão mole—semi membranosus) submetida a diferentes tratamentos térmicos. Ph.D. Thesis.

[37] Harland B.F., Oberleas D., Spiller G.A. (1993). Phytate contents of foods. CRC Handbook of Dietary Fiber in Human Nutrition.

[38] Mccrory M.A., Hajduk C.L., Roberts S.B. (2006). Procedures for screening out inaccurate reports of dietary energy intake. Public Health Nutr..

[39] Vinken A.G., Bathalon G.P., Sawaya A.L., Dallal G.E., Tucker K.L., Roberts S.B. (1999). Equations for predicting the energy requirements of healthy adults aged 18–81 y. Am. J. Clin. Nutr..

[40] Fisberg R.M., Colucci A.C.A., Morimoto J.M., Marchioni D.M.L. (2008). Food frequency questionnaire for adults from a population-based study. Rev. Saude Publica.

[41] Haubrock J., Nöthlings U., Volatier J.L., Dekkers A., Ocké M., Harttig U., Illner A.K., Knüppel S., Andersen L.F., Boeing H. (2011). Estimating Usual Food Intake Distributions by Using the Multiple Source Method in the EPIC-Potsdam Calibration Study. J. Nutr..

[42] Harttig U., Haubrock J., Knuppel S., Boeing H., EFCOVAL Consortium (2011). The MSM program: Web-based statistics package for estimating usual dietary intake using the Multiple Source Method. Eur. J. Clin. Nutr..

[43] Willett W.C., Howe G.R., Kushi L.H. (1997). Adjustment for total energy intake in epidemiologic studies. Am. J. Clin. Nutr..

[44] R Core Team (2013). R: A Language and Environment for Statistical Computing.

[45] World Health Organization (2011). Serum Ferritin Concentrations for the Assessment of Iron Status and Iron Deficiency in Populations.

[46] Agência Nacional de Vigilância Sanitária (ANVISA) (2002). Resolução 344/2002: Regulamento Técnico para a Fortificação das Farinhas de Trigo e das Farinhas de Milho com Ferro e Ácido Fólico.

[47] Allen L.H., De Benoist B., Omar D., Hurrell R. (2006). Guidelines on Food Fortification with Micronutrients.

[48] Assunção M.C.F., Santos I.S., Barro A.J., Gigante D.P., Victora C.G. (2012). Flour fortification with iron has no impact on anaemia in urban Brazilian children. Public Health Nutr..

[49] Hoppe M., Sjöberg A., Hallberg L., Hulthén L. (2008). Iron status in Swedish teenage girls: Impact of low dietary iron bioavailability. Nutrition.

[50] Associação Brasileira de Empresas de Pesquisa (ABEP) (2014). Critério Brasil 2014.

[51] Matsudo S., Araújo T., Matsudo V., Andrade D., Andrade E., Oliveira L.C., Braggion G. (2001). International physical activity questionnaire (IPAQ): Study of validity and reability in Brazil. Rev. Bras. Ativ. Fís. Saúde.

[52] World Health Organization (1995). Physical Status: The Use of and Interpretation of Anthropometry.

[53] World Health Organization (2011). Waist Circumference and Waist–Hip Ratio.

[54] Thurnham D.I., McCabe G.P. (2012). Influence of infection and inflammation on biomarkers of nutritional status with an emphasis on vitamin A and iron. Report: Priorities in the Assessment of Vitamin A and Iron Status in Populations, Panama City, Panama, 15–17 September 2010.

[55] Armah S.M., Carriquiry A.L., Reddy M.B. (2015). Total Iron Bioavailability from the US Diet Is Lower Than the Current Estimate. J. Nutr..

[56] Perignon M., Barré T., Gazan R., Amiot M.J., Darmon N. (2018). The bioavailability of iron, zinc, protein and vitamin A is highly variable in French individual diets: Impact on nutrient inadequacy assessment and relation with the animal-to-plant ratio of diets. Food Chem..

[57] Subar A.F., Freedman L.S., Tooze J.A., Kirkpatrick S.I., Boushey C., Neuhouser M.L., Thompson F.E., Potischman N., Guenther P.M., Tarasuk V. (2015). Addressing Current Criticism Regarding the Value of Self-Report Dietary Data. J. Nutr..

[58] Instituto Brasileiro de Geografia e Estatística (IBGE) (2011). Pesquisa de Orçamentos Familiares 2008–2009: Análise do Consumo Alimentar Pessoal No Brasil.

[59] Dos Santos Q., Nilson E.A., Verly E.J., Sichieri R. (2015). An evaluation of the effectiveness of the flour iron fortification programme in Brazil. Public Health Nutr..

[60] De Carvalho A.M., César C.L., Fisberg R.M., Marchioni D.M. (2014). Meat Consumption in Sao Paulo—Brazil: Trend in the Last Decade. PLoS ONE.

[61] Ribeiro M.A., Cominetti C., Kakazu M.H., Sarkis J.E., Dainty J., Fox T.E., Cozzolino S.M. (2014). Zinc absorption in Brazilian subjects fed a healthy meal. J. Hum. Nutr. Diet..

